# VEGF/Flk1 Signaling Cascade Transactivates *Etv2* Gene Expression

**DOI:** 10.1371/journal.pone.0050103

**Published:** 2012-11-19

**Authors:** Tara L. Rasmussen, Xiaozhong Shi, Alicia Wallis, Junghun Kweon, Katie M. Zirbes, Naoko Koyano-Nakagawa, Daniel J. Garry

**Affiliations:** Lillehei Heart Institute, University of Minnesota, Minneapolis, Minnesota, United States of America; University of Texas Southwestern Medical Center, United States of America

## Abstract

Previous reports regarding the genetic hierarchy between Ets related protein 71 (Er71/Etv2) and Flk1 is unclear. In the present study, we pursued a genetic approach to define the molecular cascade between Etv2 and Flk1. Using a transgenic Etv2-EYFP reporter mouse, we examined the expression pattern of Etv2 relative to Flk1 in the early conceptus. Etv2-EYFP was expressed in subset of Flk1 positive cells during primitive streak stages, suggesting that Flk1 is upstream of Etv2 during gastrulation. Analysis of reporter gene expression in Flk1 and Etv2 mutant mice further supports the hypothesis that Flk1 is necessary for Etv2 expression. The frequency of cells expressing Flk1 in Etv2 mutants is only modestly altered (21% decrease), whereas expression of the Etv2-EYFP transgenic reporter was severely reduced in the Flk1 null background. We further demonstrate using transcriptional assays that, in the presence of Flk1, the Etv2 promoter is activated by VEGF, the Flk1 ligand. Pharmacological inhibition studies demonstrate that VEGF mediated activation is dependent on p38 MAPK, which activates Creb. We identify the VEGF response element in the Etv2 promoter and demonstrate that Creb binds to this motif by EMSA and ChIP assays. In summary, we provide new evidence that VEGF activates Etv2 by signaling through Flk1, which activates Creb through the p38 MAPK signaling cascade.

## Introduction

Homozygous gene disruption of Etv2, an Ets DNA binding domain containing transcription factor (also known as Er71/Etsrp71), results in nonviable embryos at a midgestational age (E9.5). At a gross morphological level, Etv2 mutant embryos are indistinguishable from their wildtype and heterozygous littermates up to E8.5. However, the yolk sacs are markedly abnormal and histological analysis reveals that the vascular and hematopoietic lineages are absent in both the embryo and the yolk sac [1], [2], [3]. The phenotype is strikingly similar to the targeted knockouts in which a LacZ gene replaces the endogenous Flk1 gene [4]. While the Flk1 knockout embryos retain Flk1-LacZ expressing cells, the endothelial and hematopoietic lineages are lost, suggesting that Flk1^+^mesoderm is specified but does not further differentiate to form vessels or blood.

Flk1 is a cell surface receptor that is known to induce signaling cascades in response to vascular endothelial growth factor (VEGF). VEGF is known to regulate both angiogenesis and vasculogenesis through a complex series of signaling cascades. VEGF/Flk1 signaling is known to activate p38 MAPK, ERK1/2 MAPK, PKC, and other signaling cascades to induce phosphorylation of target proteins, including cellular enzymes and transcription factors [5], [6], [7]. In collaboration with Etv2 and Flk1, VEGF is essential for vasculogenesis as embryos deficient for a single VEGF allele are nonviable at a midgestational age (E10.5) and have severe vascular defects [8], [9].

The genetic hierarchy of Flk1 and Etv2 has been controversial. Initial reports have shown that Etv2 is a direct upstream regulator of Flk1 [2], [10]. Mutant embryos for Etv2 lack vessels and Flk1 expression was down-regulated [1], [2]. Overexpression of ETV2 in an ES/EB system promoted the appearance of Flk1^+^mesoderm and hematopoietic/endothelial lineages [2]. Furthermore, direct binding of ETV2 and transactivation of endothelial promoters provided supporting evidence that ETV2 can activate Flk1 expression [2], [10]. In contrast, Kataoka, et al. recently suggested that the VEGF/Flk1 signal may induce Etv2. They proposed that Flk1^+^/Pdgfra^+^primitive mesoderm requires Etv2 function to progress to Flk1^+^/Pdgfra^−^ vascular mesoderm [11]. In order to further elucidate the genetic interactions between Flk1 and Etv2, we undertook detailed gene expression studies and analysis of the compound mutants for Flk1 and Etv2. Our genetic studies demonstrate that Etv2 and Flk1 are closely linked genes that are involved in the same pathway and that Flk1 signaling is essential for the Etv2 expression. We provide data that the Flk1 receptor is activated by its ligand VEGF and activates the p38 MAPK signaling cascade, which, in turn, activates Creb to induce expression of Etv2. Together, our data and previously published data [2], [10] support the notion that Flk1 and Etv2 activate each other bidirectionally depending on the stage of mesodermal development.

## Methods

### Immunohistochemistry

A previously described Etv2-EYFP transgenic reporter mouse line [12] was used to generate stage appropriate embryos. Embryos were cryopreserved, embedded, and sectioned as previously described [12]. Embryos were staged according to Downs and Davies [13]. Sections were treated with primary antibodies for rabbit anti-VEGFR2 (1∶500 Cell Signaling 55B11), goat anti-brachyury (N-19, 1∶200 Santa Cruz sc-17743), and chicken anti-GFP (1∶500 abcam ab13970) sera. Secondary antibodies included donkey anti-chicken Dylight 488 (1∶400), donkey anti-goat Cy3, donkey anti-rabbit Cy3, and donkey anti-goat Cy5 (1∶400, Jackson ImmunoResearch) sera.

### β-galactosidase Staining

Flk1-LacZ embryos were harvested and fixed in 4% paraformaldehyde (PFA) at 4°C for 10 minutes. Embryos were then washed with PBS, transferred into staining solution (5 mM potassium ferricyanide, 5 mM potassium ferrocyanide, 1 mg/ml X-gal in PBS) and incubated at 37°C for 24 hours. The staining solution was washed from the embryos before final fixation at 4°C for one hour in 4% PFA. Fixative was removed through successive washes and embryos were imaged on a Zeiss Stereo Discovery V2.0 microscope before paraffin embedding and sectioning. Sections were counterstained with nuclear fast red using standard techniques.

### Flow Cytometry

Embryos were digested into a single cell suspension as previously described [12]. Cells were stained with APC- or PECy7-conjugated Flk1 antibody (1∶1000, eBiosciences 17-5821 or BD Pharmingen 561259, respectively) for 30 minutes on ice. Excess antibody was removed by washing with PBS. Cells were profiled on a FACS Aria (BD Biosciences).

### Quantitative PCR

Embryos were harvested at E7.75, E8.0, and E9.0 and staged using morphological assessments [13] and by somite number [14]. Embryos were either mascerated in Trizol (Invitrogen) and stored at −80°C or digested with trypsin as described above and FACS sorted directly into Trizol (Invitrogen). RNA from whole embryos was isolated according to manufacturer’s instructions. cDNA was generated from 0.5 ug total RNA using the RT Vilo Kit (Invitrogen) according to the manufacturer’s instructions. Total RNA from 400–1000 sorted cells was isolated using the PureLink Mini RNA system (Ambion) and amplified with the MessageAmp™II aRNA Amplification kit (Ambion). Expression levels were determined using FAM- (Gapdh, 4352339E) or VIC-labeled (Flk1, mm00440085_m1 and Etv2 mm01176581_g1) Taqman probes (Applied Biosystems).

### Transcriptional Assays

Promoter fragments were cloned into pGLT, a pGL3 basic vector (Promega) modified to include a TATA box [1]. Promoters were mutated or truncated using PCR and confirmed by sequencing. Identified cAMP Responsive Elements (CREs) were mutated from TGATC to TGAAA. 293T cells were maintained in Dulbecco’s modified eagle media containing 10% FBS and co-transfected with Lipofectamine 2000 (Invitrogen) according to the manufacturer’s recommendations. The pGLT promoter-luciferase reporters were co-transfected with the CMV Renilla reporter vector (internal control) and expression vectors for Flk1 (Open Biosystems clone ID 4238984) and Creb m1 (Addgene plasmid 22969) [15], as indicated in the figures and figure legends. For VEGF activation studies, the transfection cocktail was removed 18 hours post transfection and replaced with DMEM containing 5% FBS with or without VEGF 165 (293-VE, R&D Systems). For inhibitor studies, the cells were pretreated (for one hour) 17 hours after transfection with DMEM containing 5% FBS and appropriate concentrations of inhibitors [SB202190 (Sigma Aldrich S7067) and GF109203X (Sigma Aldrich B6292)] or vehicle (0.1% DMSO). Media containing inhibitor was replaced with media supplemented with or without VEGF 165 (293-VE, R&D Systems). Unless otherwise described, cells were harvested 6 hours after VEGF treatment. Cells were washed twice with PBS before treatment with lysis buffer (20% glycerol, 0.1% Triton-X 100, and 1 mM DTT in PBS supplemented with a Complete Mini protease inhibitor cocktail (Roche)). Luciferase activity was measured and standardized to Renilla Luciferase activity using the Dual Luciferase Reporter Assay System (Promega) on a Sirius Berthold Detection Systems luminometer.

### Chromatin Immunoprecipitation (ChIP)

Embryoid bodies (EBs) were maintained in differentiation medium and harvested at day 3.5. Chromatin DNA was isolated from EBs as previously described [16]. ChIPAb+CREB antibody (Millipore) and the ChIP assay kit (Millipore) were utilized to analyze the protein-DNA interaction between Creb1 and the CRE2 motif of the Etv2 promoter. Routine PCR and cyber green qPCR were performed to detect the CRE2 region or a region in Gapdh gene with specific primer pairs (CRE2 F: CTCCCCAAGTTCTTTTCCAAGC, CRE2 R: CTGATAGGGGAGGGGGAATTTT, Gapdh F: TGACGTGCCGCCTGGAGAAA, Gapdh R: AGTGTAGCCCAAGATGCCCTTCAG).

### Electrophoretic Mobility Shift Assay (EMSA)

A Creb1 expression plasmid (Addgene plasmid 22969) was utilized in the TNT Reticulocyte lysate system (Promega) according to the manufacturer’s instructions to generate in vitro translated protein [16]. A double stranded oligonucleotide harboring the CRE2 motif (5′-GACCCCCAGCTCTGAAATCTCGGAAAATTCCC-3′) was labeled with ^32^P. The protein-DNA complex was analyzed on a 4% TBE non-denaturing gel, and imaged using a Typhoon phosphorImager Model (GE Healthcare) [17]. The cold probes harboring the wild type CRE2 motif or the mutated CRE2 motif (5′-GACCCCCAGCTCTGAAATCTCGGAAAATTCCC-3′) were preincubated with the protein before addition of the radioactive probe in the competition assays. Anti-Creb1 (Santa Cruz sc186) or rabbit IgG sera was added to the protein-DNA complex for the supershift assay.

## Results

### Etv2 is Coexpressed with Flk1

**Figure 1:**
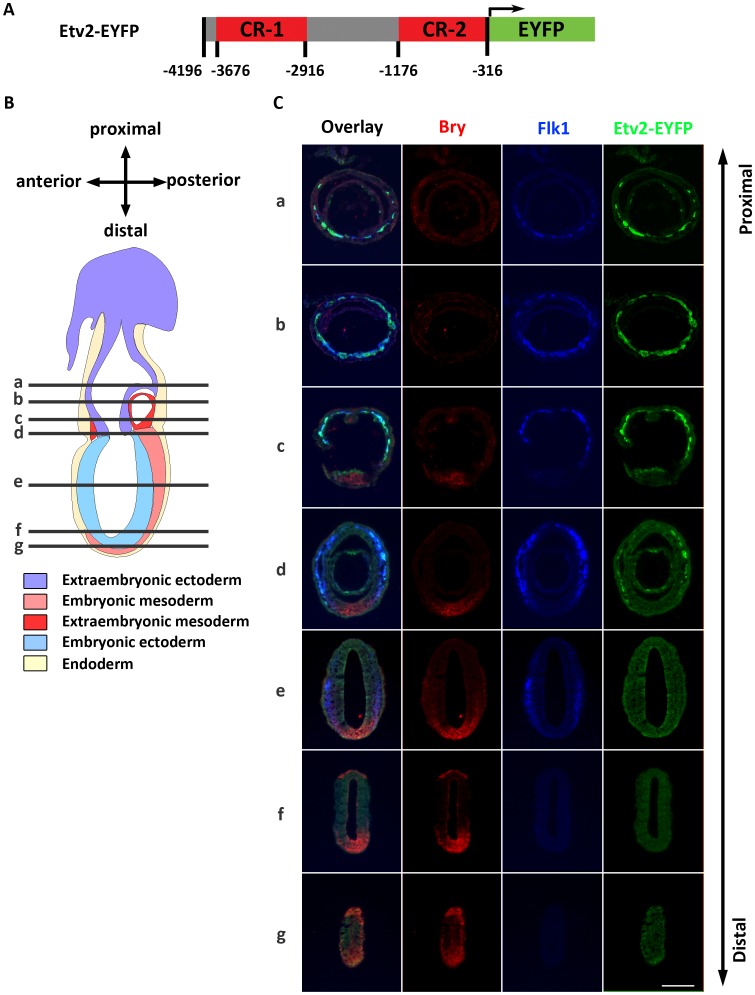
Flk1 expression precedes Etv2 expression during embryogenesis. (A) Diagram of the transgenic construct used in this study. Numbers shown identify the genomic location relative to the translation start site of Etv2. CR-1 represents conserved region one and CR-2 represents conserved region two. (B) Schematic diagram of the embryonic axes and germ layers of a late streak stage embryo. Black lines indicate approximate levels of sections in C. (C) A series of transverse sections of a late streak stage embryo. Representative sections were stained with antibodies to Brachyury (Bry), Flk1 and EYFP (scale bar: 100 microns).

**Figure 2:**
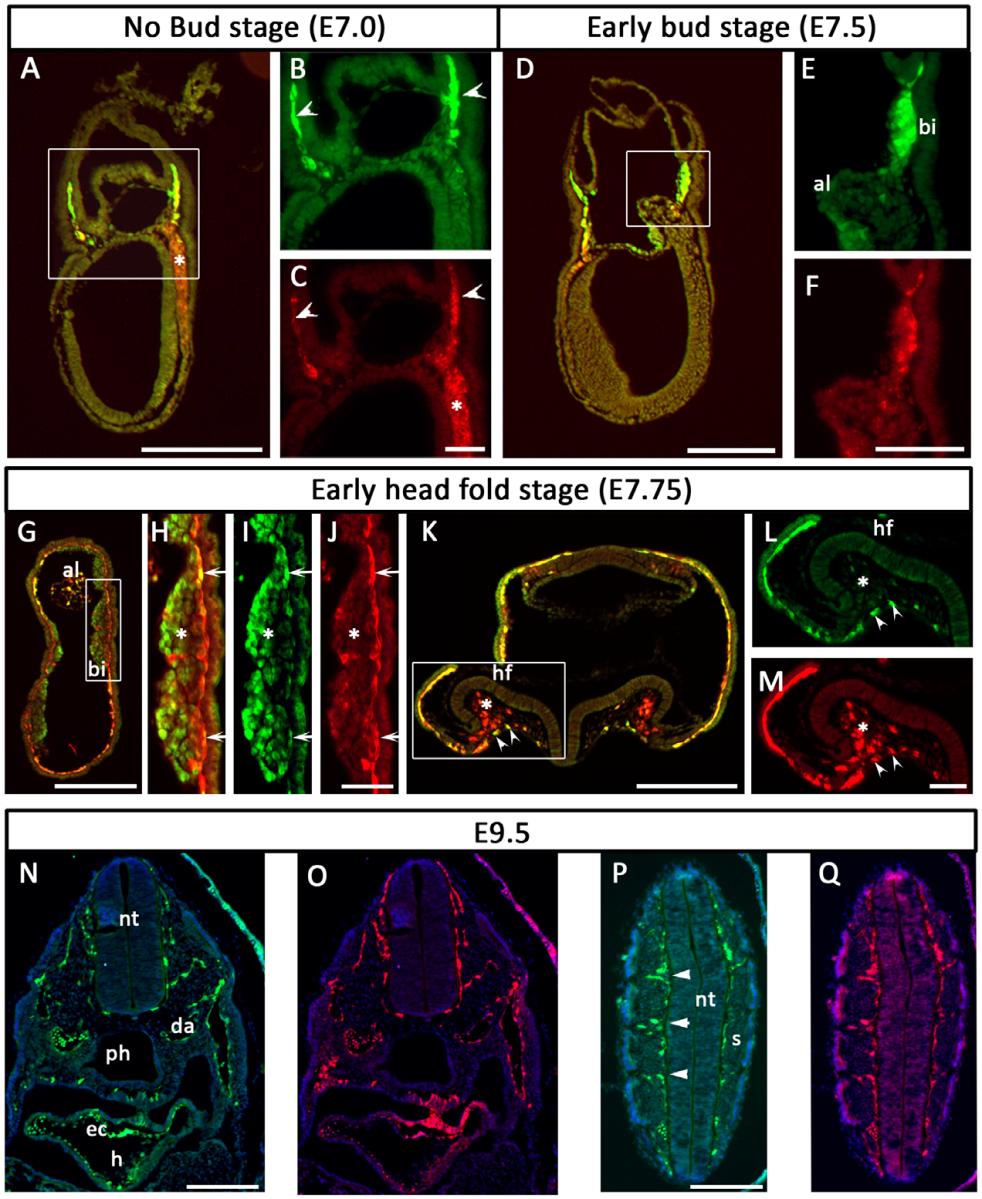
Etv2 is coexpressed with Flk1. (A–Q) Expression analysis of Etv2-EYFP and Flk1 in E7.0 to E9.5 embryos. Sections were stained with the GFP antibody (A, B, D, E, G–I, K, L, N, P; shown in green) or the Flk1 antibody (A, C, D, F–H, J, K, M, O, Q; shown in red). DAPI nuclear staining is shown in blue (N–Q). Yellow indicates overlap of green and red channels (A, D, G, H, K). A–C: No bud stage. D–F: Early-bud stage. G–M: Early head-fold stage. N–Q: E9.5. The boxed area in A is enlarged in B–C; the boxed area in D is enlarged in E–F; the boxed area in G is enlarged in H–J, and the boxed area in K is enlarged in L–M. Arrowheads in B, C point to progenitors of blood islands coexpressing Flk1 and EYFP. Arrows and arrowheads in H–M indicate endothelial lineages co-expressing Flk1 and EYFP. Asterisks in A, H–M indicate regions in which either gene is expressed alone. Arrowheads in P indicate developing intersomitic vessels. Structures are designated as follows (al: allantois, bi: blood island, da: dorsal aorta, h: heart, ec: endocardium, hf: head fold, nt: neural tube, ph: pharynx, s: somite). Bars in A, D, G, K, N, and P indicate 200 µm. Bars in C, F, J, and M indicate 50 µm.

Previous studies have identified a 3.9 kb sequence upstream of the Etv2 gene that, when fused to LacZ or EYFP reporters, recapitulates expression of endogenous Etv2 [1], [12]. We used the Etv2-EYFP transgenic reporter (Fig. 1A) to examine the expression pattern of Etv2, particularly in comparison to Flk1 expression. First, we examined a series of transverse sections at the late streak stage (E6.5–E6.75) (Fig. 1B, C). Brachyury antibody was used to mark the primitive streak. Flk1 was undetectable in the primitive streak but was expressed in the mesoderm of the embryo proper emerging from the Brachyury positive mesodermal progenitors (Fig. 1Cd, e). Etv2-EYFP was expressed in the subset of Flk1 positive cells in the embryo proper (Fig.1Cd, e). Flk1 was also expressed in the extraembryonic mesodermal cells, which are progenitors of yolk sac vasculature and blood islands (Fig. 1Ca, b, c). In this population, Flk1 was coexpressed with Etv2. At the no bud stage (E7.0) in a parasagittal section, we observed that Etv2-EYFP and Flk1 expression largely overlapped in the extraembryonic mesoderm (Fig. 2A, B, C, arrowheads). However, in the mesoderm of the embryo proper most cells expressed Flk1 alone (Fig. 2A, B, C, asterisk) and few cells co-expressed Flk1 and EYFP. By the early bud stage (E7.5), Etv2 and Flk1 were coexpressed in the extra-embryonic mesoderm, including the outer layer of the developing blood islands (Fig. 2D, E, F). At the early head fold stage (E7.75), Etv2 and Flk1 were coexpressed in the allantois (Fig. 2G) and the endothelial layer of the extraembryonic mesoderm (Fig. 2G, Hi, J, K, L, M) as well as the endocardial and vascular progenitors (Fig. 2K, L, M). In contrast, hematopoietic cells within the blood islands expressed Etv2 alone (Fig. 2G, H, I, J, asterisk) and paraxial mesoderm of the head fold expressed Flk1 alone (Fig. 2K, L, M, asterisk). At E9.5 Flk1 and Etv2-EYFP were coexpressed in the committed vascular cells, such as the endocardium (Fig. 2N,O), dorsal aorta (Fig. 2N,O), and intersomitic vessels (Fig. 2P,Q, arrowheads). Previous studies have demonstrated that cells migrate out of the primitive streak into the embryonic and extraembryonic mesoderm towards anterior and proximal directions to form the first vascular and hematopoietic structures [18], [19]. Based on this information, the presence of Flk1 single positive cells in the mesoderm of gastrulating embryos close to the primitive streak (Figs 1Bd,e, 2A), and later coexpression with Etv2 in the extraembryonic mesoderm (Figs 1Ba, b, c, 2A, B, C, D, E, F, G, H, I, J, K, L, M) as well as in the vascular structures (Fig. 2N, M, O, P, Q) suggests that Flk1 expression precedes that of Etv2 during mesodermal differentiation. To further analyze the relative timing of the onset of gene expression, we analyzed younger embryos at early and mid streak stages. Early streak embryos, expressing few Brachyury^+^cells, expressed neither Flk1 nor Etv2-EYFP (SI Fig. 1B). Mid streak embryos clearly expressed Flk1 (SI Fig. 1Cd, e, f, g). However, detection of Etv2 at this stage was inconclusive (SI Fig. 1). Therefore, we conclude that initiation of Flk1 and Etv2 expression occurs in a very tight window at or after the mid streak stage.

### Flk1 Expression is Modestly Affected in Etv2 Mutant Embryos

**Figure 3:**
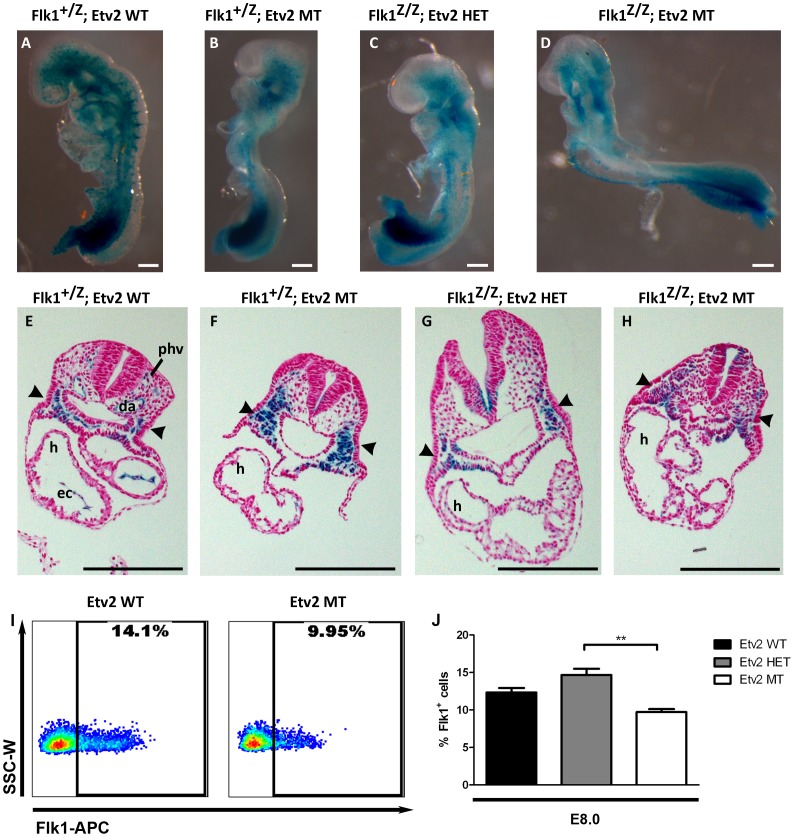
Etv2 mutant embryos phenocopy Flk1 null embryos. (A–D) Whole mount β-galactosidase staining of Flk1 LacZ allele in a representative Etv2 wildtype, Flk1 heterozygous embryo (A); Etv2 mutant, Flk1 heterozygous embryo (B); Etv2 heterozygous, Flk1 null embryo (C); and Etv2 mutant, Flk1 null embryo (D). (E–H) Histological sections of β-galactosidase stained embryos counterstained with nuclear fast red in Etv2 wildtype, Flk1 heterozygous embryo (E); Etv2 mutant, Flk1 heterozygous embryo (F); Etv2 heterozygous, Flk1 null embryo (G); and Etv2 mutant, Flk1 null embryo (H). (I–J) FACS analysis of Flk1 stained Etv2 wildtype and mutant embryos. Representative FACS plots are shown (I) and data from all experiments are graphically represented (J) (SSC-W: side scatter- width; **p<0.01). Scale bars represent 200 µm. Arrowheads represent lateral plate mesoderm. Structures are designated as follows (h: heart; ec: endocardium; da: dorsal aorta; phv: primary heart vein).

Using mouse genetics, the finding that a single gene mutation results in a haploinsufficient phenotype is rare. Often, when genes have a close molecular interaction, breeding single allele mutations together to form compound heterozygotes will generate an enhanced phenotype by compounding the effects of two haploinsufficient genes. To examine if Etv2 and Flk1 have such an interaction, we bred the heterozygous Etv2 mutant mice [1] to the heterozygous Flk1-LacZ knock-in knock-out mice [4]. Animals heterozygous for both the Etv2 mutation and the Flk1-LacZ allele were viable, were obtained at the expected ratios, and were indistinguishable from the wildtype controls. Thus, we further crossed the compound heterozygotes to each other to determine if compound null embryos have an enhanced phenotype. The compound Flk1 null, Etv2 mutant embryos (Flk1^Z/Z^-Etv2 MT, Fig. 3D) died at the same stage as the single mutant embryos (Flk1^+/Z^-Etv2 MT and Flk1^Z/Z^-Etv2 Het, Fig. 3B, C) and appeared morphologically similar to the single gene disruptions.

We took advantage of the Flk1-LacZ reporter present in the Flk1 null allele. As previously reported in otherwise wildtype embryos, Flk1-LacZ is expressed in a clearly endothelial expression pattern (Fig. 3A) and the Flk1 mutant embryos retain Flk1-LacZ expression, but have a diffuse expression pattern in whole mount embryos (Fig. 3C) [4]. Based on previous reports that Etv2 is an upstream activator of Flk1 [2], [10], we expected that Etv2 mutants heterozygous for the Flk1-LacZ allele would have reduced or absent LacZ expression. However, we observed similar levels of LacZ in the Etv2 mutant as we did in the Flk1 mutant (Fig. 3A, B, C). Furthermore, we also detected similar amounts of LacZ expression in the Etv2 and Flk1 compound mutant (Fig. 3D). These observations support the notion that Etv2 is not required for Flk1 gene activation.

Histological analysis revealed that Flk1-LacZ is expressed in the vascular structures of the Flk1-LacZ heterozygous; Etv2 wildtype embryos (Fig. 3E). LacZ expression was also observed in the lateral plate mesoderm (Fig. 3E, arrowheads). In all mutant genotypes, the vascular structures were absent and LacZ expression was primarily localized to the lateral plate mesoderm (Fig. 3F, G, H). We predict that these LacZ positive cells represent mesodermal progenitors that failed to differentiate into the typical lateral plate mesoderm lineages, such as endothelial and hematopoietic lineages. Importantly, compound Etv2 and Flk1 null embryos do not appear to have an enhanced phenotype. Thus, Etv2 and Flk1 knockouts are neither synergistic nor additive, and their respective individual and compound mutants are phenocopies. This suggests that removing either of these genes disables the same developmental process.

As previous reports [2], [10] demonstrated that Etv2 activates Flk1 expression, the lack of a reduction of the Flk1-LacZ reporter activity in the Etv2 mutants (Fig. 3B, F) was an unexpected finding. However, recent reports have also begun to question the dependence of Flk1 expression on Etv2 [11], [12]. Thus, we used FACS analysis of Etv2 wildtype and mutant embryos at E8.0 to verify the extent to which Flk1 expression is affected (Fig. 3I, J). We observed that the number of cells expressing Flk1 was reduced by 21% compared to the wildtype embryos, but Flk1 positive cells were clearly present (Fig. 3I, J). These results demonstrated that the initiation of Flk1 expression is not dependent on Etv2 expression and can occur in the absence of Etv2.

### Etv2 Expression is Dependent on Flk1

**Figure 4:**
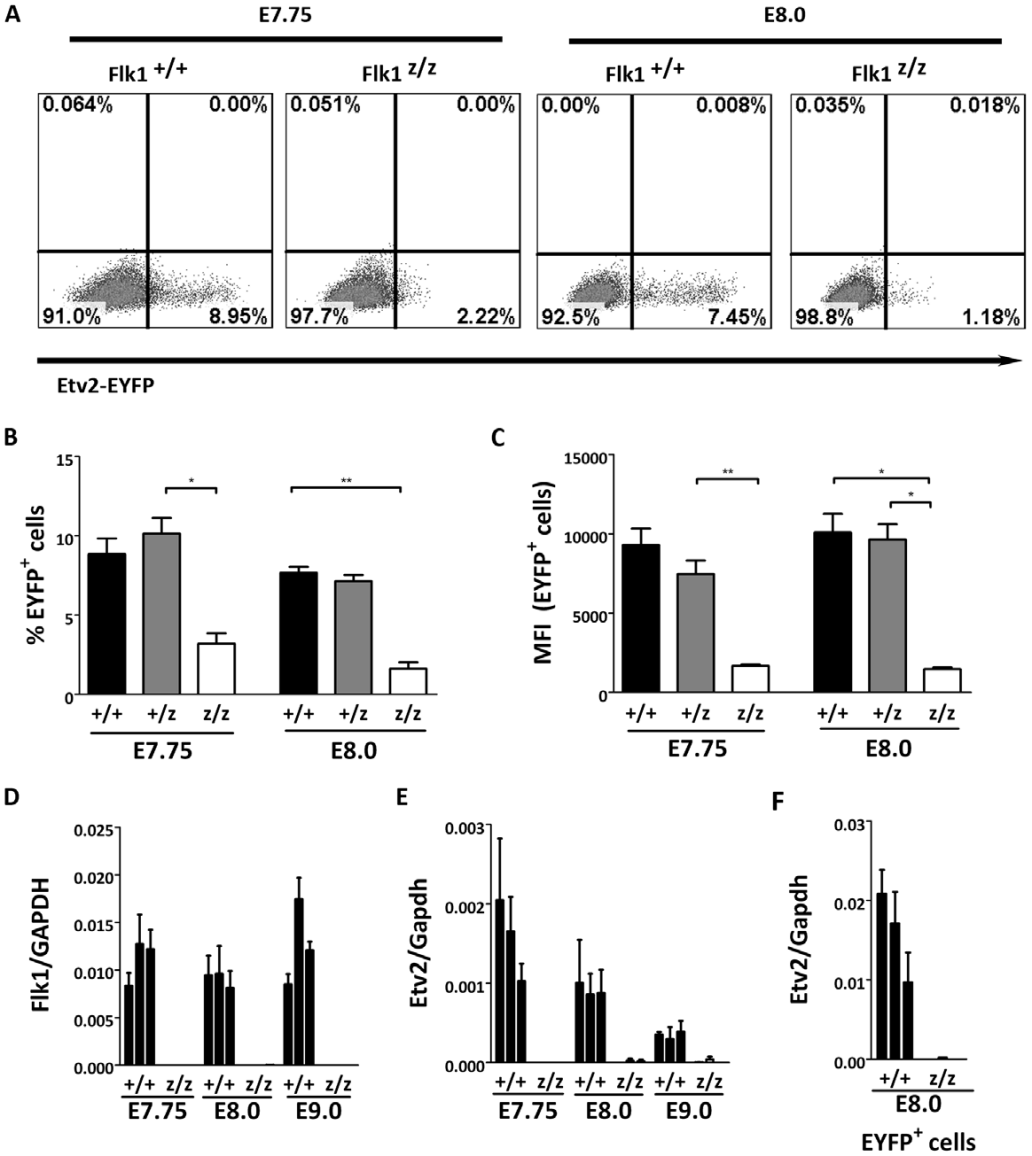
Etv2 is absent in Flk1 null embryos. (A–C) FACS analysis of the Etv2-EYFP transgenic reporter crossed into the Flk1 wildtype and mutant backgrounds at E7.75 and E8.0. Representative FACS profiles are shown (A). (B–C) Data from all experiments are compiled graphically to show the percent of EYFP positive cells in Flk1 wildtype, heterozygous, and null embryos (B) and the mean fluorescent intensity of the EYFP positive cells (C). (D–E) Quantitative PCR for Flk1 (D) and Etv2 (E) was performed on RNA isolated from Flk1 wildtype and null embryos at E7.75, E8.0, and E9.0. (F) Quantitative PCR for Etv2 was performed on FACS isolated Etv2-EYFP positive cells. Each bar represents triplicate measurements from independent samples (single embryos). Asterisks indicate the following: **p<0.01, *p<0.05.

To further analyze the genetic interaction between Etv2 and Flk1, we crossed the Etv2-EYFP transgenic reporter into the Flk1 null background. Unlike the wildtype littermate controls, EYFP expression in Flk1 null embryos was undetectable through fluorescent microscopy of whole mount embryos or in immunohistochemically stained embryo sections (data not shown). However, by FACS we were able to detect a small population of cells that express EYFP (Fig. 4A, B). The number of cells expressing EYFP was significantly lower in mutants compared to wildtype controls at E7.75 and E8.0 (Fig. 4A–B). The amount of EYFP expressed per cell was also dramatically decreased in Flk1 mutant embryos at E7.75 and E8.0 compared to age matched wildtype controls (Fig. 4A,C). The severe reduction of the EYFP reporter activity suggests that Etv2 is either minimally expressed or not expressed in the Flk1 null embryos and further suggests that Etv2 expression is dependent on the presence of Flk1. One possible explanation is that in the Flk1 null embryos, the appropriate progenitor population is absent and that the lack of Etv2 reporter is due to the absence of this pool of cells. However, Flk1-LacZ positive progenitors are present (Fig. 3) and therefore, we explored the possibility that Etv2 was downstream of a Flk1 signalling cascade.

To further support this possibility, we quantified gene expression in Flk1 wildtype and null embryos by qRT-PCR. Flk1 was expressed at similar levels at E7.75, E8.0 and E9.0 in wildtype embryos, and was absent in Flk1 mutants at all ages (Fig. 4D). Etv2 expression was highest at E7.75 and declined over two days in Flk1 wildtype embryos, whereas it was below the detection threshold in the Flk1 null embryos at all stages (Fig. 4E). In order to verify whether Etv2 was indeed absent, equal numbers of EYFP^+^cells (750) were sorted from Flk1 wildtype and mutant embryos at E8.0. After RNA amplification, qRT-PCR analysis was performed. In wildtype embryos, this enriched the Etv2 transcript 10–20 fold relative to Gapdh and compared to whole embryos (Fig. 4E,F). However, Etv2 transcript was still below the detection threshold in Flk1 null embryos. These data indicate that, although Etv2-EYFP promoter is weakly activated in the Flk1 mutants, no detectable Etv2 transcript is present. It should be noted that this low level of EYFP expression was only detectable by FACS, but not by immunohistochemistry. Therefore, we conclude that Etv2 expression is absent in Flk1 null embryos from E7.75 to E9.0.

### The Etv2 Promoter Responds to VEGF in the Presence of Flk1

**Figure 5:**
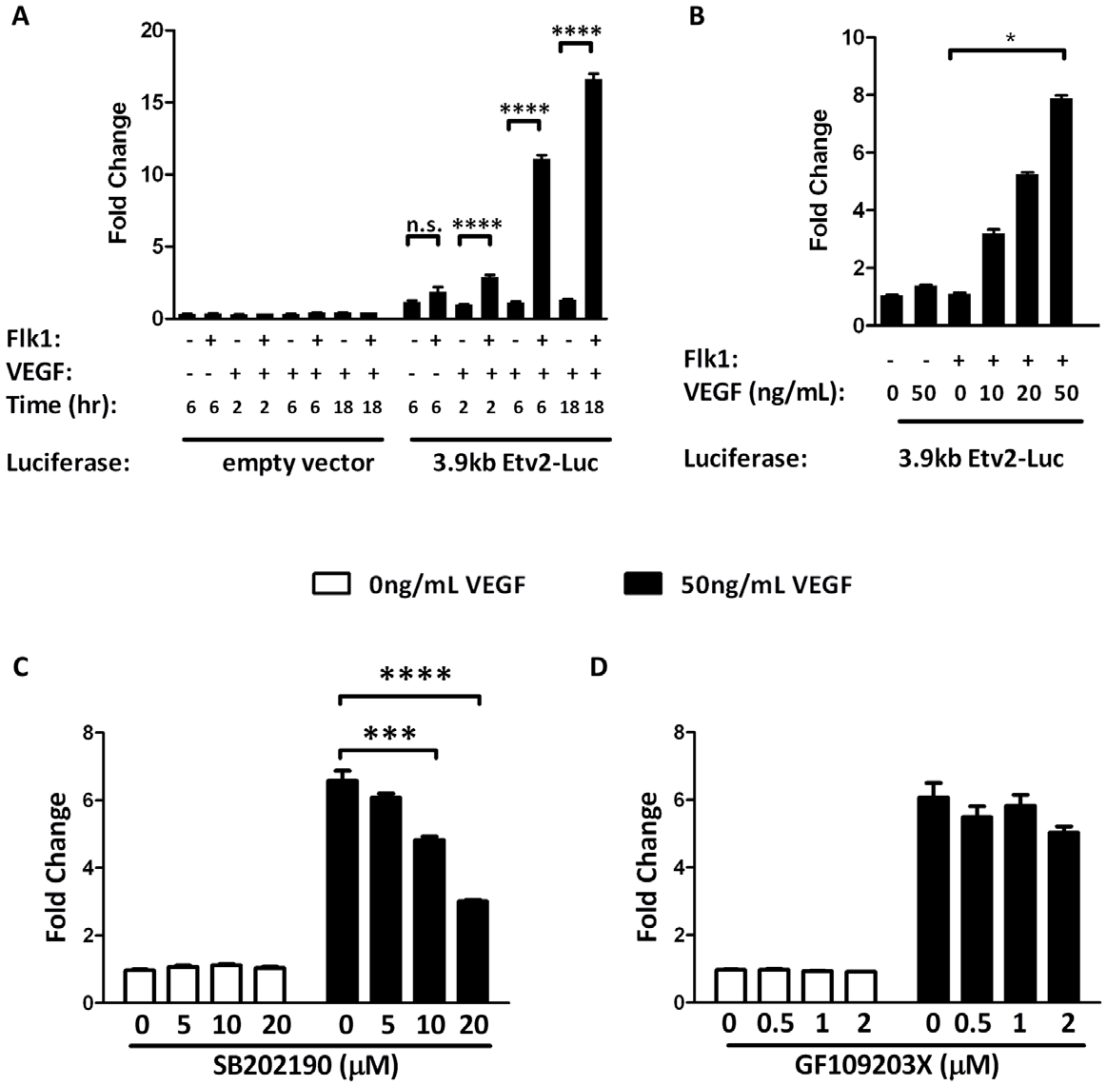
VEGF activates the Etv2 promoter. (A–B) Transcriptional assays using an empty luciferase vector or the 3.9 kb Etv2 luciferase reporter standardized to the CMV-Renilla Luciferase reporter. Flk1 cDNA or a balancer DNA plasmid is co-transfected and the cells are treated with or without VEGF (50 ng/ml) for 2–18 hours as indicated in the panel (A). Flk1 or a balancer DNA is cotransfected and cells are treated for 6 hours with varying amounts of VEGF (0–50 ng/ml) as indicated in the panel (B). (C–D) Transcriptional inhibition assays in which Flk1 cDNA and the 3.9 kb Etv2 Luciferase promoter are co-transfected followed by pretreatment of various doses of SB202190 (C), or GF109203X (D) and a 6 hour treatment of 50 ng/ml VEGF. Data were analyzed by two-way anova (A,C,D) or one-way anova (B). n.s.: not significant; *: p<0.05; ***: p<0.001; ****: p<0.0001.

Next, we examined whether Etv2 is activated by the Flk1 signaling pathway. Flk1 is known to function as a receptor for VEGF. To determine whether Etv2 is downstream of this signaling pathway, we examined the effect of Flk1 and VEGF on the 3.9 kb Etv2 luciferase reporter in 293T cells. Flk1 was introduced by cotransfection of the Flk1 expression vector and VEGF was administered by adding a recombinant protein to the culture. The 3.9 kb Etv2 Luciferase reporter showed very low baseline activity, as expected in a non-endothelial cell line (Fig. 5A). Adding Flk1 alone, VEGF alone, or Flk1 and VEGF in combination for 2, 6, or 18 hours had no effect on the empty luciferase vector (Fig. 5A). However, cotransfection with the Flk1 expression vector and incubation of 293T cells with VEGF for 2, 6, or 18 hours resulted in activation of the luciferase reporter by as much as 15 fold. Activation increased with longer VEGF exposure times (Fig. 5A). This effect is specific to the 3.9 kb regulatory region since it was not observed in the empty vector controls. Furthermore, VEGF treatment had a dose dependent activation of the 3.9 kb Etv2 luciferase reporter construct (Fig. 5B).

VEGF is known to signal through downstream signaling pathways including p38 MAPK and PKC [5], [6], [7]. We used inhibitors for p38 MAPK (SB202190) and PKC (GF109203X) to determine whether either of these pathways are critical for VEGF activation of Etv2. Following transfection (17 hours), cells were pretreated for one hour with media containing pharmacological inhibitors. Because continuous treatment with the vehicle alone (0.1% DMSO) had negative effects on VEGF activation, the inhibitor containing media was replaced with VEGF containing media after the pre-treatment. In this experimental design, SB202190, a p38 MAPK inhibitor inhibited VEGF-mediated activation in a dose-dependent manner (Fig. 5C). These data demonstrate that p38 signaling through VEGF contributes to Etv2 activation. In contrast, GF109203X, a PKC inhibitor, had no effect on the VEGF activation of the Etv2 luciferase reporter (Fig. 5D). Together, these data suggest that p38 MAPK acts positively in the Flk1 signaling cascade to regulate transcription of Etv2.

### Creb1 Binds to the CRE2 Motif in the Etv2 Promoter

**Figure 6:**
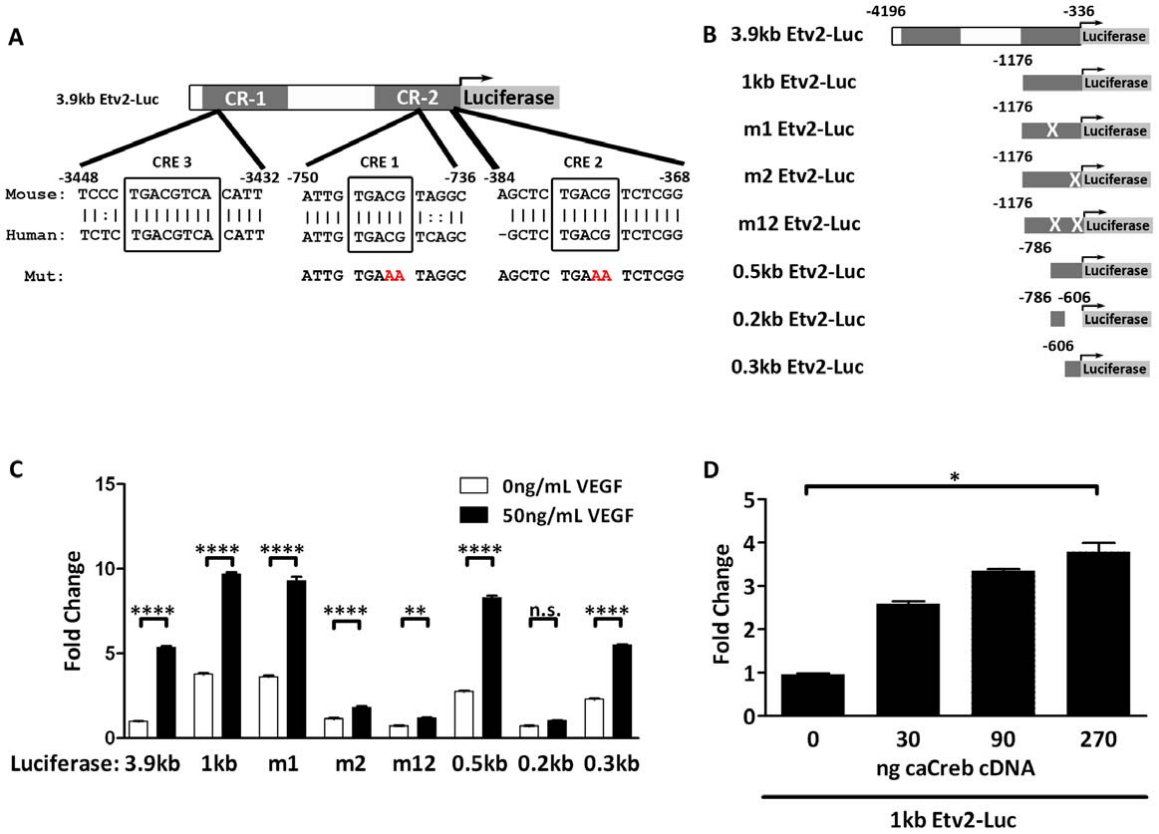
VEGF activation of the Etv2 promoter is dependent on a Creb binding site. (A) A schematic of the 3.9 kb Etv2 luciferase reporter showing 3 evolutionary conserved Creb binding motifs (CRE1-3) and the mutations schematized in panel B. Numbers reflect genomic position relative to the translational start site of Etv2. (B) A schematic of the truncation and mutation strategy. × indicates a CG to AA mutation of either CRE1 or CRE2. Numbers indicate genomic position relative to the translational start site of Etv2. (C) Transcriptional assays using the constructs shown in B co-transfected with Flk1 cDNA and treated with or without VEGF. (D) Transcriptional assay using the 1 kb Etv2 luciferase cotransfected with increasing amounts of expression vector encoding constitutively phosphorylated Creb cDNA. Data were analyzed using two-way anova (C) or Kruskal-Wallis nonparametric t-test with Dunn’s post test comparison (D). n.s.: not significant; *: p<0.05; **: p<0.01; ****: p<0.0001.

We analyzed the Etv2 promoter using dcode (dcode.org) and rVista for evolutionarily conserved motifs that are known to be downstream of VEGF/Flk1/p38 MAPK signaling. We identified three evolutionarily conserved CREs (c-AMP response elements) which are known to bind to CRE binding proteins (Creb) (Fig. 6A). Two of the CREs were in the Conserved Region 2 (CR-2), at −745 to −741 and −378 to −374 from the translation initiation site. The third CRE was found in Conserved Region 1 (CR-1) at −3436 to −3443 from the translation initiation site. Interestingly, Creb has been shown to be downstream of VEGF and is activated by both PKC and p38-MAPK pathways [6]. Therefore, in order to address whether one or more of the CREs were involved in activation of Etv2 by VEGF signaling, we generated a number of modified Etv2-luciferase constructs (Fig. 6B). First, we truncated the 3.9 kb promoter to the 1 kb region, leaving CR-2. We verified that this region was responsive to VEGF activation (Fig. 6C). Mutation of the CRE 1 (m1) within this 1 kb region had no effect on baseline expression or VEGF activation. Mutation of CRE 2 (m2) resulted in a statistically significant VEGF activation, however, this mutation reduced the baseline expression level and the VEGF activation was dramatically reduced compared to the 1 kb or m1 mutation constructs. The combination of m1 and m2 (m12) further reduced the ability of VEGF to activate the *Etv2* gene. We engineered additional constructs including the proximal 0.5 kb regulatory region, which harbors both CRE 1 and CRE 2. This construct retains VEGF responsiveness; however, the 0.2 kb region which contains CRE 1 is not activated by VEGF. Finally, the 0.3 kb region that harbors CRE 2 is sufficient to respond to VEGF. In conclusion, our analyses demonstrate that the 0.3 kb region that harbors CRE 2 is sufficient for the activation of Etv2 transcription by VEGF and an intact CRE 2 binding site is critical for this regulation (Fig. 6C). Finally, we examined whether this reporter can be activated by Creb. Cotransfection of a vector encoding a constitutively phosphorylated form of Creb1 activated the 1 kb Etv2 luciferase reporter in a dose dependent fashion (Fig. 6D).

### Creb1 Binds CRE 2

**Figure 7:**
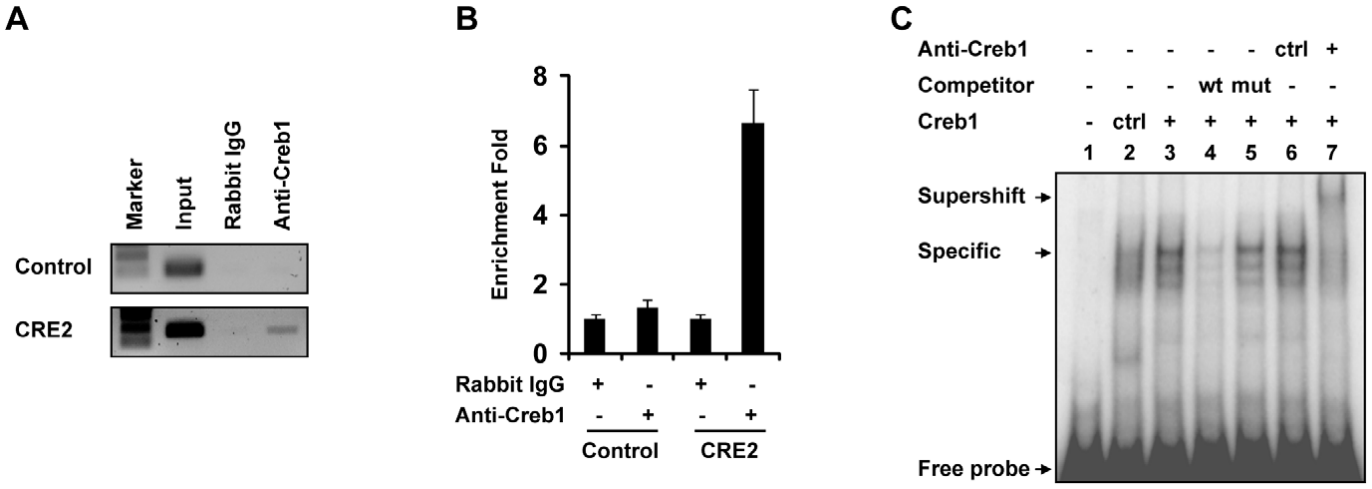
Creb1 binds to the CRE2 motif in the Etv2 promoter. (A–B) Creb1 specifically interacts with the CRE2 motif in the Etv2 promoter as shown by ChIP assay. EBs were collected 3.5 days after initiating mesoderm differentiation. A region in the Gapdh gene was used as a negative control (Control) and 1% of the total chromatin DNA before the immunoprecipitation was used as a positive control (Input). The PCR products were run on a gel for direct visualization (A) and qPCR was performed for quantitation (B). (C) Creb1 binds CRE2 directly *in vitro*. Creb1 was synthesized *in vitro*. The oligonucleotides harboring CRE2 motif is labeled with 32P. Synthesized Creb1, non-radioactive oligonucleotides, and the antibodies were incubated with radioactive oligonucleotide probes as indicated in the figure. The interaction between Creb1 and the CRE2 motif was analyzed on a 4% TBE gel.

Next, we determined whether Creb1 is capable of binding to the CRE 2 motif. We performed a ChIP assay using embryoid bodies (EBs) differentiated for 3.5 days. We chose this system because under mesoderm inducing conditions, EBs contain abundant endothelial cells and Etv2, Flk1, and Creb1 are highly expressed (data not shown). A Gapdh control motif is present in the input lane, but is not enriched when pulled down with the Creb1 antibody compared to the nonspecific IgG antibody. The CRE 2 motif is pulled down specifically with the Creb1 antibody but not with the IgG control (Fig. 7A). Quantification of the precipitates by qPCR showed that CRE 2 motif was enriched seven fold with the specific antibody over the IgG control antibody (Fig. 7B). Finally, we performed an EMSA to examine whether a synthesized Creb1 protein binds to a radioactive CRE2 oligonucleotide (Fig. 7C). We observed a low level of background signal in the control protein lysate (Fig. 7C, lane 2), presumably due to the endogenous Creb1 protein or other Creb family members in the rabbit reticulocyte lyaste. However, the Creb1-CRE2 interaction is specific as it is repressed by the cold probe harboring the wild type CRE2 motif (Fig. 7C, lane 4), but not the mutated CRE2 motif (Fig. 7C, lane 5), and the protein-DNA complex is supershifted by the Creb1 antibody (Fig. 7C, lane 7), but not by the control antibody (Fig. 7C, lane 6). In summary, our results demonstrate that Creb1 binds to CRE2 both in vivo and in vitro.

## Discussion

Etv2 and Flk1 are both essential for endothelial and hematopoietic lineage development. To date, there has not been a conclusive study that determines genetic hierarchy of these genes. Two reports place Etv2 upstream of Flk1 and propose an Etv2-Flk1 hierarchical cascade. We and others [11], [12] have recently begun to question this hierarchy, as we demonstrated Flk1 expression is not as significantly down regulated in Etv2 mutants as previously reported. We have systematically analyzed the relationship between these two genes and have redefined this hierarchy. We provide three major findings that support this overall hypothesis. First, we defined that the initiation and maintenance of Flk1 expression is independent of Etv2 gene expression through E8.5. Second, we determined that VEGF activates Etv2 through the Flk1 receptor and the p38-MAPK signaling pathway, and our third discovery was that a 300 base pair region of the Etv2 promoter, which harbors the Creb binding motif, was sufficient for activation of Etv2 by VEGF.

First, we demonstrated that onset of Etv2 expression is dependent on Flk1 expression in vivo. While this seemingly conflicts with previous biochemical reports that Etv2 is upstream of Flk1 [2], [10] and that the requirement for Etv2 precedes Flk1 expression [20], recent reports from our group and Nishikawa’s laboratory demonstrate that initial expression of Flk1 takes place in the absence of Etv2 [11], [12]. Waering et al. report that Etv2 functions before or at the same time as Flk1 expression using the conditional deletion of Etv2 by Flk1-Cre knock-in mouse model. However, it has been shown that expression of similar Flk1 gene constructs (GFP and LacZ) is limited mainly to the endothelial lineage because of the interference by the neomycin cassette [21]. Therefore, although their study suggests that Etv2 and Flk1 function at similar developmental stages, it does not preclude the possibility that Flk1 is required to activate Etv2 expression. Using genetic labeling methods and immunohistochemistry, we showed that Flk1 is expressed more broadly than Etv2 expression in embryonic mesoderm and is initiated during the same developmental stage, that Etv2 is absent in Flk1 deficient embryos, and that Flk1 is present in Etv2 deficient embryos. These discoveries indicate that Flk1 signalling activates Etv2 in the initiation of hematopoiesis and vasculogenesis in gastrulating embryos. This finding is consistent with the notion that hematopoietic and endothelial lineages arise as a subpopulation of the Flk1^+^mesoderm. The expression patterns of Etv2 and Flk1 further suggest that Etv2 may transcriptionally maintain or reinforce Flk1 expression in a context dependent manner, which would explain the previously published results that Etv2 binds to and activates the Flk1 promoter and that overexpression of Etv2 leads to an increase in Flk1^+^mesoderm [2], [10].

Secondly, we determined that VEGF/Flk1 signaling activates Etv2 in a p38-MAPK dependent fashion by phosphorylating and activating Creb1. Previous studies have focused on BMP, Wnt, and Notch as upstream regulators of Etv2 [2]. Interestingly, these studies show that Flk1 and Etv2 are both activated by BMP, Wnt and Notch. While the authors concluded that Etv2 activates Flk1, they do not exclude the possibility that Flk1 also activates Etv2. It would be interesting to further investigate the mechanism in which these developmental pathways affect and interact with the Flk1-Etv2 signaling cascade and the regulation of hemogenic lineages. Here, we provide evidence that Etv2 and Flk1 act not only in the same genetic cascade, but also function in the same signaling cascade. We observed that VEGF and Flk1 activate Etv2 in non-endothelial cell lineages, suggesting that this is an over-arching mechanism that would also be active in the uncommitted mesodermal progenitor cell. We also observed that a p38 MAPK inhibitor reduced the ability of VEGF to activate the Etv2 promoter. Etv2 is expressed transiently in a narrow developmental window (E7–E9.5), whereas VEGF signaling is sustained into adulthood and is known to induce many physiological cell changes. Not surprisingly, transduction of VEGF signals is complex and involves many signaling cascades, including PKC and ERK-MAPK pathways. It is logical to think that the transduced VEGF signal would be affected in a context dependent way and may rely on the balance of signaling pathways. While our studies highlight the signals that activate Etv2 gene expression, we recognize that a potent transcriptional repressor may override the VEGF/Flk1-p38 MAPK-Creb activation and extinguish or significantly attenuate Etv2 expression.

Finally, we identified a 300 bp region of the Etv2 promoter which is sufficient for VEGF activation. This region harbors an evolutionary conserved CRE motif which is essential for VEGF activation. Furthermore, Creb1 binds to this site and a constitutively phosphorylated (activated) Creb1 activates the Etv2 promoter in a dose dependent fashion. Recent studies have identified Creb as a direct upstream regulator of Etv2 [22], but as a downstream effector of PKA signaling as opposed to Flk1/Vegf. While the CRE binding sites identified are the same and the Yamashita group shows that mutation of these sites prevents luciferase reporters from responding to PKA, we show that mutation of these sites prevents a response from VEGF. Similar to our studies, the Yamashita group also undertook ChIP and EMSA studies to show that Creb binds to the Etv2 promoter. However, they show that Creb only binds to the Etv2 promoter with forced expression of PKA 1.5 days after differentiation, whereas in the present study we show that Creb1 binds to the Etv2 regulatory region in unperturbed D3.5EBs (the stage in which we see peak Etv2 expression). We have further identified that a 300 bp minimal promoter is sufficient to activate Etv2 in the presence of VEGF signals in non-endothelial cells. Further studies are warranted to determine whether this region is sufficient to drive expression in an endothelial expression pattern in vivo and whether this regulatory motif would be sufficient to down regulate expression at the appropriate developmental stage to recapitulate endogenous Etv2 expression.

In conclusion, we show that VEGF signals through Flk1 in a p38 MAPK dependent pathway to activate Creb1, which activates Etv2. These findings significantly advance the field by defining the hierarchy of genes involved in initiating vasculogenesis and outlining the mechanism in which Etv2 is transcriptionally activated.

## Supporting Information

Figure S1 **Flk1 and Etv2 expression is initiated during the midstreak stage.** (A) Schematic diagram of the embryonic axes and germ layers of early and mid streak stage embryos. Black lines indicate approximate levels of sections in B and C. (B) A series of transverse sections of an early streak stage embryo. Representative sections were stained with antibodies to Brachyury (Bry), Flk1 and EYFP (scale bar: 20 microns). (C) A series of transverse sections of a midstreak stage embryo. Representative sections were stained with antibodies to Flk1 and EYFP (scale bar: 20 microns).
(TIF)
