# Supplementary figures and images for: VEGF/Flk1 Signaling Cascade Transactivates Etv2 Gene Expression
Source: PLoS One. 2012 Nov 19;7(11):e50103. doi: 10.1371/journal.pone.0050103 (PMC3501484; doi:10.1371/journal.pone.0050103)

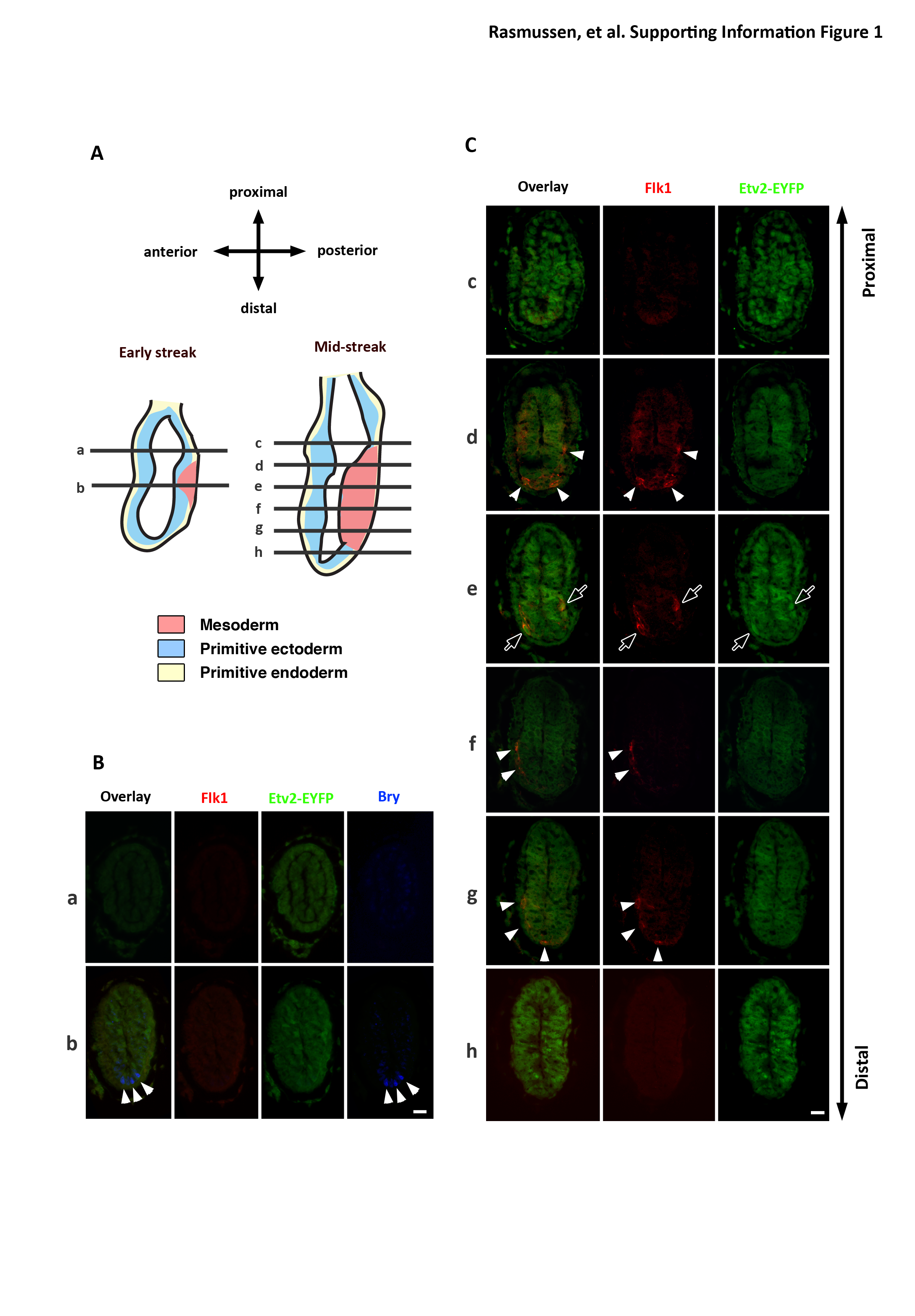

Supplement: Figure S1 — Flk1 and Etv2 expression is initiated during the midstreak stage. (A) Schematic diagram of the embryonic axes and germ layers of early and mid streak stage embryos. Black lines indicate approximate levels of sections in B and C. (B) A series of transverse sections of an early streak stage embryo. Representative sections were stained with antibodies to Brachyury (Bry), Flk1 and EYFP (scale bar: 20 microns). (C) A series of transverse sections of a midstreak stage embryo. Representative sections were stained with antibodies to Flk1 and EYFP (scale bar: 20 microns). (TIF) [file pone.0050103.s001.tif]
